# Bone morphogenetic protein-7 attenuates pancreatic damage under diabetic conditions and prevents progression to diabetic nephropathy via inhibition of ferroptosis

**DOI:** 10.3389/fendo.2023.1172199

**Published:** 2023-05-24

**Authors:** Sang Hyun Song, Dawool Han, Kyeonghui Park, Jo Eun Um, Seonghun Kim, Minhee Ku, Jaemoon Yang, Tae-Hyun Yoo, Jong In Yook, Nam Hee Kim, Hyun Sil Kim

**Affiliations:** ^1^ Department of Oral Pathology, Yonsei University College of Dentistry, Seoul, Republic of Korea; ^2^ Oral Cancer Research Institute, Yonsei University College of Dentistry, Seoul, Republic of Korea; ^3^ R&D Center, MET Life Science, Seoul, Republic of Korea; ^4^ Department of Radiology, Yonsei University College of Medicine, Seoul, Republic of Korea; ^5^ Convergence Research Center for Systems Molecular Radiological Science, Yonsei University, Seoul, Republic of Korea; ^6^ Department of Internal Medicine, Yonsei University College of Medicine, Seoul, Republic of Korea

**Keywords:** Bmp7, ferroptosis, TGF-β, fibrosis, diabetic nephropathy

## Abstract

**Background:**

Approximately 30% of diabetic patients develop diabetic nephropathy, a representative microvascular complication. Although the etiological mechanism has not yet been fully elucidated, renal tubular damage by hyperglycemia-induced expression of transforming growth factor-β (TGF-β) is known to be involved. Recently, a new type of cell death by iron metabolism called ferroptosis was reported to be involved in kidney damage in animal models of diabetic nephropathy, which could be induced by TGF-β. Bone morphogenetic protein-7 (BMP7) is a well-known antagonist of TGF-β inhibiting TGF-β-induced fibrosis in many organs. Further, BMP7 has been reported to play a role in the regeneration of pancreatic beta cells in diabetic animal models.

**Methods:**

We used protein transduction domain (PTD)-fused BMP7 in micelles (mPTD-BMP7) for long-lasting *in vivo* effects and effective *in vitro* transduction and secretion.

**Results:**

mPTD-BMP7 successfully accelerated the regeneration of diabetic pancreas and impeded progression to diabetic nephropathy. With the administration of mPTD-BMP7, clinical parameters and representative markers of pancreatic damage were alleviated in a mouse model of streptozotocin-induced diabetes. It not only inhibited the downstream genes of TGF-β but also attenuated ferroptosis in the kidney of the diabetic mouse and TGF-β-stimulated rat kidney tubular cells.

**Conclusion:**

BMP7 impedes the progression of diabetic nephropathy by inhibiting the canonical TGF-β pathway, attenuating ferroptosis, and helping regenerate diabetic pancreas.

## 1 Introduction

Diabetic nephropathy (DN) is a major complication of diabetes and a causative factor of renal dysfunction; glomerulosclerosis, hypertrophy of basement membrane, extracellular matrix (ECM) accumulation, tubulointerstitial fibrosis, tubular cell damage, and cell death are well-known pathologic changes of DN. Approximately 30% of patients with diabetes develop DN; however, the biological mechanism of DN pathogenesis remains unclear. Previous studies demonstrated that changes in renal hemodynamics, hyperglycemia-induced oxidative stress, an inflammatory response, and enhanced renin-angiotensin-aldosterone system activity were involved in the pathogenesis of DN (1, 2). Recently, TGF-β1 has come to be viewed as the main regulator and therapeutic target of kidney fibrosis and tubular cell death, given its upregulation in patients with diabetes (3–7). Since the apoptosis or necroptosis of podocytes and renal tubular cells have been extensively studied (8), we focused on ferroptosis, which is emerging as a cause of cell death in many diseases including cancer and metabolic disease.

Ferroptosis is a newly proposed type of cell death characterized by iron-dependent lipid oxidation. The key players of ferroptosis are cysteine transporter and glutathione peroxidase 4 (GPX4); low intracellular cysteine levels result in reduced glutathione synthesis and the dysfunction of lipid oxidative damage repair, leading to ferroptosis (9, 10). Emerging evidence has revealed that ferroptosis is involved in acute kidney injury (AKI) and diabetic nephrology (11–13), including its pathogenesis. TGF-β1-stimulates tubular cells; erastin-stimulated tubular cells and streptozotocin (STZ)-induced diabetic mice show similar ferroptotic conditions in the kidney (14).

Multiple studies have reported that bone morphogenetic protein-7 (BMP7) effectively inhibits fibrosis involving various organs, such as retinopathy, liver fibrosis, and peritoneal fibrosis (15–17). In diverse renal disease models, BMP7 has been found to counteract fibrosis by suppressing the activation of multiple signaling pathways including the p38, p44/42 MAPK, and TGF-β pathways, as well as by reducing reactive oxygen species (ROS) formation (18–20). In an STZ-induced animal diabetes model, BMP7 was found to protect against diabetic kidney injury, counteracting TGF/SMADs signaling (20, 21). Further, several studies have identified the role of BMP7 in supporting β-cell regeneration and differentiation in adult and prenatal pancreas. For example, BMP7 plays a critical role during the transdifferentiation of ductal progenitor cells into exocrine beta cells. These results suggest the therapeutic potential of BMP7 for treating DN by protecting renal fibrosis as well as including pancreatic exocrine regeneration.

Since recombinant human BMP (rhBMP) undergoes rapid clearance and enzymatic degradation *in vivo* (22), we designed a micellar formulation of protein transduction domain (PTD)-BMP7 (mPTD-BMP7) to overcome the pharmacodynamic limitation of rhBMP7 in animal experimental models and to enhance endosomal transduction *in vitro* and *in vivo* (23, 24). We investigated whether mPTD-BMP7 ameliorated pancreatic damage in STZ-induced diabetes and attenuated diabetes-induced ferroptotic deterioration of the kidney.

## 2 Materials and methods

### 2.1 Cell culture and treatment of NRK-52E cells

Immortalized rat proximal tubular epithelial cells, NRK-52E ( CRL-5171, ATCC, VA, USA), were cultured in Dulbecco’s modified Eagle’s medium (DMEM) supplemented with 5% fetal bovine serum (FBS) and 1% penicillin-streptomycin-amphotericin B. Subconfluent NRK-52E cells were starved in FBS-free condition for 24 hours; then, the medium was replaced with DMEM with 0.5% FBS. For the TGF-β1 group, TGF-β1 (10 ng/mL; R&D Systems, Minneapolis, MN, USA) was added to the culture, and erastin (Selleckchem) was used as the positive control for ferroptosis induction. Both groups were treated with mPTD-BMP7 (250 ng/mL). AGEs (advanced glycation end-products) were obtained through the non-enzymatic reaction of BSA (Sigma-Aldrich) and glycerolaldehyde as described by Valencia et al. (25). Briefly, sterile filtered 30% BSA solution was incubated with 70 mM glycolaldehyde dimer in sterile PBS without calcium chloride and magnesium chloride for 3 days at 37°C. After incubation, the AGEs were dialyzed against sterile PBS to remove remaining glycolaldehyde at 4°C using 10 kDa cut-off cassettes. Control BSA was prepared *via* the same process without reaction with glycolaldehyde dimer. Concentration of dialyzed BSA-AGEs was determined by BCA assay and extent of AGEs modification was confirmed by fluorescence and absorbance as described by Valencia et al. (25). After starvation in FBS-free condition for 24 hours, the medium was replaced with 0.5% FBS and 500 μg/mL BSA-AGEs or BSA-control and further incubated for 72 hours.

### 2.2 Animal experiments

Eight-week-old male C57BL/6 mice were injected with either the diluent (control; n=5) or streptozotocin (STZ; Sigma-Aldrich; 50 mg/kg; n=20) intraperitoneally for 5 days consecutively. To confirm diabetes mellitus (DM; fasting blood glucose >300 mg/dL), tail vein blood glucose was measured, and only 13 mice were diagnosed with DM. Five mice from the control group and seven from the DM group were treated with the vehicle (10 μg) intraperitoneally every 72 h. The other six mice from the DM group were treated with mPTD-BMP7 (10 μg) intraperitoneally every 72 h. After 16 weeks, the mice were anesthetized with Zoletil (10 mg/kg; Virbac, Carros, France), and their kidneys and pancreas were extracted. Body weight, kidney weight, and levels of blood glucose, HbA1c, serum bilirubin, serum creatinine, blood urea nitrogen (BUN), 24 h urinary albumin, and creatinine were determined at the time of sacrifice. Blood glucose level was measured using a glucometer, and HbA1c level was measured using a glycohemoglobin analyzer. Then, 24-h urinary albumin was assessed *via* the enzyme-linked immunosorbent assay (ELISA; Nephrat II, Exocell Inc., Philadelphia, PA, USA), and total bilirubin and 24-h urinary creatinine levels were analyzed using a Cobas 8000 C502 analyzer (Roche, Mannheim, Baden Württemberg, Germany). Insulin (NBP2-62853, Novus Biologicals, Littleton, CO, USA) and C-peptide (NBP2-82124, Novus Biologicals, Littleton, CO, USA) concentrations were determined by ELISA.

### 2.3 Quantitative real-time polymerase chain reaction

Total RNA was isolated from the cells, whole pancreas, and kidney samples using Trizol (Invitrogen), and the cDNA was synthesized using a reverse transcriptase premix product (AccuPower CycleScript RT PreMix, Bioneer). Transcript levels were detected using the 7300 Real-Time PCR system (Applied Biosystems) with TB Green^®^ Premix Ex Taq TM II (Takara, Shiga, Japan). mRNA expression level was calculated using △CT values and the results are shown as relative expression normalized by that of the housekeeping genes (*r18s, Actb, Rpl13a*). The primer sequences used in this study are presented in **Tables 1**, **2**.

**Table 1:** Primer sequences used in the qPCR analysis of the animal experiment.

Gene	Species	Accession number	Primer sequences from 5’ to 3’
Tgfb1	Mus musculus	NM_011577	Forward GCTTCAGCTCCACAGAGAAGA
			Reverse GACAGAAGTTGGCATGGTAGC
Col1a1	Mus musculus	NM_007742	Forward GTCAGACCTGTGTGTTCCCT
			Reverse TCCATCGGTCATGCTCTCTC
Ccn2	Mus musculus	NM_010217	Forward AAGAAGGGCAAAAAGTGCATC
			Reverse CGCAGAACTTAGCCCTGTATG
Fn1	Mus musculus	NM_010233	Forward AGAAGTTTGTGCATGGTGTCC
			Reverse ACTTGGACAGGTCCAGTTGTG
Gpx3	Mus musculus	NM_008161	Forward ACCAATTTGGCAAACAGGAG
			Reverse TCTTTCTCCCCGTTCACATC
Gpx4	Mus musculus	NM_008162	Forward CCGGCTACAATGTCAGGTTT
			Reverse ACGCAGCCGTTCTTATCAAT
Slc7a9	Mus musculus	NM_021291	Forward GGATTCCTCTGGTGACCGTA
			Reverse GGACTACCCAAGATGCTGGA
Fth1	Mus musculus	NM_010239	Forward GAGAAGAGCCGAGACAATGG
			Reverse GAGCCTAAGCCTGAATGCAC
Hspb1	Mus musculus	NM_013560	Forward ACTGGCAAGCACGAAGAAAG
			Reverse AGGGAAGAGGACACTAGGGT
Ins	Mus musculus	NM_008387	Forward AGCGTGGCTTCTTCTACACAC
			Reverse CTGGTGCAGCACTGATCTACA
Pdx1	Mus musculus	NM_008814	Forward CGCGAATATCCTCCTGAAAG
			Reverse CCTGTTGGCAAAGAATGGTT
Prox1	Mus musculus	NM_008937	Forward TGAATCCCCAAGGTTCAGAG
			Reverse AAAGGCATCATGGCATCTTC
Krt19	Mus musculus	NM_008471	Forward GGTCAGTGTGGAGGTGGATT
			Reverse CCTCAATCCGAGCAAGGTAG
Slc2a1	Mus musculus	NM_011400	Forward GAGGCCACCTGAGAGTGTTC
			Reverse AGTGCCTAGCCTTGGACTGA
Syp	Mus musculus	NM_009305	Forward TGGAAATTCAACCCCAAGAG
			Reverse CCTTTTAGGCTCCACATCCA
Actb	Mus musculus	NM_007393	Forward CACCATGTACCCAGGCATTG
			Reverse CACACAGAGTACTTGCGCTC
Hnf1b	Mus musculus	NM_009330	Forward CCACCCCCATGTTAAGACAC
			Reverse AATGGGAGGCTTCCTGAGAT
Neurod1	Mus musculus	NM_010894	Forward TCAGCATCAATGGCAACTTC
			Reverse AAGATTGATCCGTGGCTTTG
Cxcr4	Mus musculus	NM_009911	Forward GAAACTGCTGGCTGAAAAGG
			Reverse CTGTCATCCCCCTGACTGAT
Rpl13a	Mus musculus	NM_009438	Forward GAGGTCGGGTGGAAGTACCA
			Reverse TGCATCTTGGCCTTTTCCTT

**Table 2:** Primer sequences used in qPCR analysis of the cell experiment.

Gene	Species	Accession number	Primer sequences from 5’ to 3’
Tgfb1	Rattus norvegicus	NM_021578	Forward TGAGTGGCTGTCTTTTGACG
			Reverse TGGGACTGATCCCATTGATT
Tgfbr1	Rattus norvegicus	NM_012775	Forward TGAGTGGCTGTCTTTTGACG
			Reverse TGGGACTGATCCCATTGATT
Col1a1	Rattus norvegicus	NM_053304	Forward TTCTGAAACCCTCCCCTCTT
			Reverse CCACCCCAGGGATAAAAACT
Ccn2	Rattus norvegicus	NM_022266	Forward AGAGTGGAGATGCCAGGAGA
			Reverse CACACACCCAGCTCTTGCTA
Fn1	Rattus norvegicus	NM_019143	Forward GTGGCTGCCTTCAACTTCTC
			Reverse TTGCAAACCTTCAATGGTCA
Gpx3	Rattus norvegicus	NM_022525	Forward GAGAAGAGCCGAGACAATGG
			Reverse GAGCCTAAGCCTGAATGCAC
Gpx4	Rattus norvegicus	NM_017165	Forward CCGGCTACAATGTCAGGTTT
			Reverse ACGCAGCCGTTCTTATCAAT
Slc7a9	Rattus norvegicus	NM_053929	Forward CAGGGGGTGAGTACCCCTAT
			Reverse TAAAAGGCCGCACACACATA
Fth1	Rattus norvegicus	NM_012848	Forward ATGATGTGGCCCTGAAGAAC
			Reverse TCATCACGGTCAGGTTTCTG
Hspb1	Rattus norvegicus	NM_031970	Forward CTGGTGTCCTCTTCCCTGTC
			Reverse GCTCCAGACTGTTCCGACTC
18s	Rattus norvegicus	NR_042637	Forward CAAGTAGGAGAGGAGCGAGC
			Reverse CATGTCTAAGTACGCACGGC

### 2.4 Measurement of lipid peroxidation

The level of malondialdehyde (MDA) was measured using a lipid peroxidation assay kit (Abcam, Cambridge, United Kingdom). For live cell imaging, the Image-iT^®^ lipid peroxidation kit (Thermo Fisher Scientific) was used. For the MDA assay, cultured cells were homogenized in MDA lysis buffer with 5% butylated hydroxytoluene, and the harvested kidney tissues were homogenized in distilled water with 5% butylated hydroxytoluene, followed by the addition of 2 N perchloric acid. After centrifugation of samples at 13,000 × g for 10 min at 4°C, the supernatant of each sample was collected, and MDA levels were measured using thiobarbituric acid, which reacts with MDA to produce a compound that has maximum absorbance at a wavelength of 532 nm. For the live cell analysis of lipid peroxidation, cells were seeded in four-well chambers (3 × 10^4^ per well) and pretreated with mPTD-BMP7 for 16 h before TGF-β1 or erastin stimulation. The Image-iT^®^ lipid peroxidation sensor was added into each well and incubated for 30 min at 37°C. The culture medium was removed, and samples were washed with phosphate-buffered saline (PBS). Images were acquired using an LSM700 confocal microscope (Carl Zeiss Vision, Hallbergmoos, Germany) under ×40 magnification. Fluorescence intensity was quantified using the publicly available ImageJ program.

### 2.5 Glutathione assay

Glutathione concentration (reduced form of glutathione only) was measured using a glutathione assay kit (Sigma-Aldrich). Cultured cells and harvested kidney tissues were lysed in glutathione buffer, and 5% sulfosalicylic acid was added later. The cells were centrifuged at 700 × g for 5 min and kidney samples were centrifuged at 8000 × g for 10 min; the supernatants were collected. NADPH-generating mix was added to the glutathione reaction buffer and incubated for 10 min at room temperature (RT) to generate NADPH. Then, 20 μL of supernatant and 160 μL of NADPH solution were mixed and incubated for 10 min at RT. Glutathione concentrations were determined by the reaction with 5,5’-dithio-bis-(2-nitrobenzoic acid) at a wavelength of 415 nm.

### 2.6 Immunohistochemistry

Serial paraffin sections were stained with hematoxylin and eosin (H&E) for routine morphological observation. For immunohistochemical (IHC) staining, tissue sections were deparaffinized with xylene, rehydrated in serially diluted ethyl alcohol, and immersed in 3% hydrogen peroxide. Antigen retrieval was carried out by microwave heating in 10-mM citric acid buffer (pH 6.0) for 20 min. Nonspecific binding of protein was blocked with 2.5% goat serum (MP-7451, Vector Laboratories) for 30 min at RT, followed by overnight incubation at 4°C with a primary antibody. After washing with PBS, the sections were incubated with a secondary antibody, horseradish peroxidase-conjugated goat anti-rabbit IgG (MP-7451, Vector Laboratories), for 30 min at RT. After washing with PBS, 3,3’ diaminobenzidine was added as the chromogen and the color developed was detected using Envision Detection Systemic of Agilent (K5007, Agilent). Hematoxylin was used for counter staining, followed by mounting. The primary antibodies used were as follows: anti-4-hydroxynonenal (4-HNE; NBP2-59353, Novus Biologicals), anti-MDA (NBP2-59367, Novus Biologicals), and anti-fibronectin (ab2413, abcam), anti-GPX4 (ab125066, abcam), anti-CTGF (ab209780, abcam), and anti-Collagen Ia (ab21286, abcam). Quantitative analysis of IHC and Sirius red staining was evaluated using five randomly taken images of renal cortex per subject. For each image, the positive signal was extracted through the color threshold process *via* the ImageJ program (open source software), and the final value was the average of multiplying the intensity and area of signal of five renal cortex images.

### 2.7 Statistical analysis

All data are representative of at least three independent experiments. Statistical differences were analyzed using paired t-test and Mann–Whitney test using Prism 5 (GraphPad, San Diego, CA). A P value < 0.05 was considered statistically significant.

## 3 Results

### 3.1 mPTD-BMP7 abrogates TGF-β1- and erastin-induced kidney tubular cell damage

**Figure 1:**
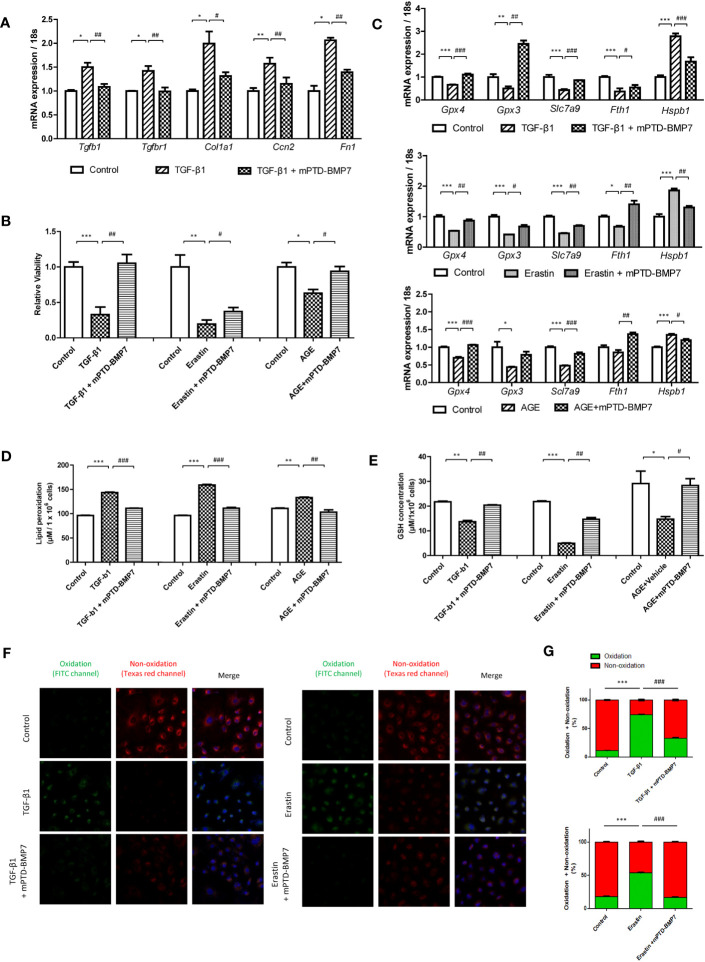
mPTD-BMP7 attenuates TGF-β1- or erastin- or AGEs-induced cellular damage in NRK-52E cells. **(A)** mPTD-BMP7 significantly ameliorates the expression of fibrosis-related downstream genes in transforming growth factor-beta1 (TGF-β1)-stimulated NRK-52E cells after 96 h. **(B)** Salvage from cell death by the administration of mPTD-BMP7 is observed on the JuLi real-time live cell analyzer (NanoEntek). NRK-52E cells were stimulated by TGF-β1 or erastin or AGEs. **(C)** The increase in the mRNA levels of ferroptosis-related genes in TGF-β1- or erastin- or AGEs-stimulated NRK-52E cells is halted after mPTD-BMP7 treatment. **(D)** Administration of mPTD-BMP7 significantly attenuates the increase in lipid peroxidation in TGF-β1- or erastin- or AGEs-stimulated NRK-52E. **(E)** Administration of mPTD-BMP7 led to significant recovery of reduced glutathione (GSH) levels in TGF-β1- or erastin- or AGEs-stimulated NRK-52E. **(F)** Increased lipid peroxidation assessed using Image-iT^®^ in TGF-β1- or erastin-stimulated NRK-52E. **(G)** Quantitative analysis of image-iT^®^ shows a significant decrease in lipid peroxidation after mPTD-BMP7 administration. (*P < 0.05; **P < 0.01; ***P < 0.001; versus control group. #P < 0.05; ##P < 0.01; ###P < 0.001; versus TGF-β1 or erastin or AGEs group).

To assess the previously known antagonizing impact of BMP7 on TGF-β1, the downstream genes of TGF-β1 were further evaluated. The expression levels of Tgfb1 and Tgfbr1 were significantly higher in the TGF-β1-stimulated NRK-52E cells that were stimulated for 96 h, followed by a significant increase in the expression of the downstream genes (Cola1, fibronectin, and Ccn2). The upregulation of fibrosis-related genes was alleviated by mPTD-BMP7 (**Figure 1A**). Next, we investigated the effect of mPTD-BMP7 on TGF-β1- and erastin-induced kidney tubular cell death. Cell viability was significantly reduced in TGF-β1- and erastin-treated tubular cell, but it was attenuated by mPTD-BMP7 (**Figure 1B**). Several key molecules of ferroptosis were evaluated to confirm whether TGF- β1 induces ferroptosis in renal tubular cell. Reduction of mRNA levels of glutathione peroxidase (Gpx3, Gpx4) and cysteine transporter (Slc7a9) were significantly attenuated by mPTD-BMP7 in TGF-β1-stimulated NRK-52E cells. Furthermore, the TGF-β1-induced changes in the mRNA levels of ferritin heavy chain (Fth1) and heat shock protein beta-1(Hspb1) were abrogated with mPTD-BMP7 treatment. NRK-52E cells stimulated with erastin, a ferroptosis inducer, showed similar findings as TGF-β1-stimulated cells (**Figure 1C**). Reduced glutathione (GSH) level was significantly reduced in TGF-β1 and erastin-stimulated conditions; however, the concentration increased with mPTD-BMP7 treatment. The increase in lipid peroxidation caused by TGF-β1 was attenuated by administration of mPTD-BMP7 at 2.5 μg/mL. In addition, mPTD-BMP7 abrogated lipid peroxidation in erastin-stimulated NRK-52E cells (**Figures 1D, E**). Taken together, these findings suggest that TGF- β1-induced tubular cell injury is involved in ferroptosis; further, mPTD-BMP7 is presumed to attenuate ferroptotic cell damage. When the same experiments were performed with advanced glycation end-products (AGEs), levels of which are elevated in hyperglycemic conditions, a similar tendency was observed as with erastin and TGF-β1, but in less degree (**Figures 1B–E**). To determine whether mPTD-BMP7 reduced lipid peroxidation of TGF-β1- and erastin-treated tubular cell, the level of lipid peroxidation was assed. Once oxidized, the fluorescence shift form a non-oxidative form (Texas red channel) to an oxidative form (FITC channel). The oxidative fluorescence intensity was increase in TGF-β1- and erastin-treated tubular cell. mPTD-BMP7 significantly reduced the process of red fluorescence to green fluorescence (**Figures 1F, G**). These results suggest that ferroptosis in renal tubular cell increased responding to highly elevated levels of TGF- β1, erastin and AGEs in diabetic condition.

### 3.2 mPTD-BMP7 attenuates pancreatic damage under diabetic conditions

**Figure 2:**
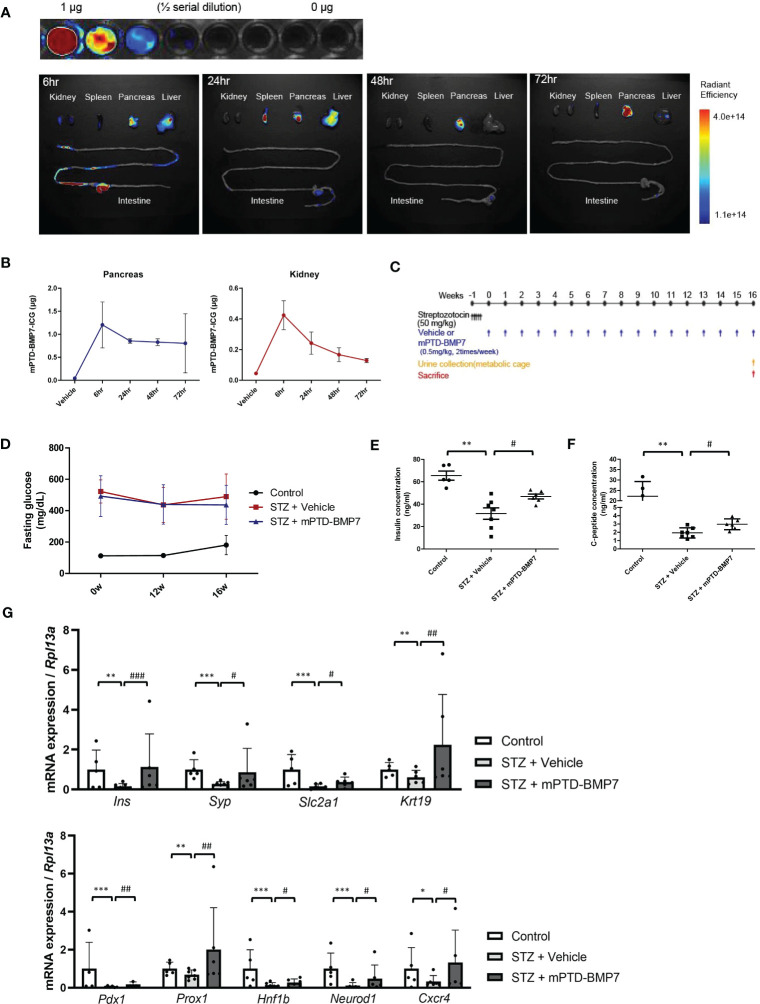
Effect of intraperitoneal administration of mPTD-BMP7 on pancreas function. **(A)** Ten micrograms of indocyanine green (ICG)-labeled mPTD-BMP7 was injected intraperitoneally, and the dissected major organs were assessed by ex vivo fluorescence imaging over time. **(B)** Quantitative analysis of the ex vivo fluorescence images of the pancreas and kidney. **(C)** Experimental schematics of streptozotocin (STZ)-induced diabetic mice. The diabetic condition was induced by an intraperitoneal injection of STZ (50 mg/kg) for 5 days consecutively, followed by an intraperitoneal injection of mPTD-BMP7 (0.5 mg/kg) twice per week. **(D)** Fasting glucose level was assessed over time for 16 weeks. Concentrations of insulin **(E)** and C-peptide **(F)** were measured in the extracted serum; these are reduced after mPTD-BMP7 administration. **(G)** mRNA levels of adult pancreatic markers (upper) and pancreatic developmental markers (lower) in STZ mice were attenuated after mPTD-BMP7 administration. (*P < 0.05; **P < 0.01; ***P < 0.001; versus control group. #P < 0.05; ##P < 0.01; ###P < 0.001; versus mPTD-BMP7 group).

To study the distribution of mPTD-BMP7, we covalently conjugated indocyanine green to mPTD-BMP7 and administered this compound to the mice *via* intra-peritoneal injection (**Figure 2A**). An ex vivo fluorescence analysis showed that a considerable amount of mPTD-BMP7 had accumulated in the pancreas at 6 h and persisted even at 72 h (**Figure 2B**). Based on the results of quantitative analysis of the ex vivo images of the pancreas and kidney, mPTD-BMP7 was administered twice a week to STZ-induced diabetic mice (**Figure 2C**). In the animal model of diabetes, the kidney/body weight, levels of blood glucose and serum bilirubin, and urine albumin-creatinine ratio (UACR) had significantly increased, these factors, especially UACR and bilirubin, showing overall improvements with mPTD-BMP7 treatment (*P*<0.05; **Table 3**). Fasting glucose (**Figure 2D**) and HbA1c levels (**Table 3**) showed little attenuation with mPTD-BMP7 treatment, whereas the concentrations of serum insulin and C-peptide showed significant reduction in diabetic mice (**Figures 2E, F**). To determine whether mPTD-BMP7 affected pancreas regeneration, we examined the qPCR result of a representative pancreas marker in diabetic pancreatic tissue. Among the pancreatic developmental markers, mRNA levels of pancreatic duodenal homeobox 1 (PDX1), which is a β-cell putative progenitor, adult pancreatic markers (**Figure 2G**, upper), and pancreatic developmental markers (**Figure 2G**, lower) severely decreased in the diabetic mice but improved significantly with mPTD-BMP7 treatment.

**Table 3:** Effect of mPTD-BMP7 assessed by clinical parameters in the animal models of diabetes.

	Control (n=5)	STZ + Vehicle (n=7)	STZ + mPTD-BMP7 (n=6)
Body weight (g)	28.97 ± 0.63	21.09 ± 1.24	21.57 ± 2.26
Kidney weight (mg)	155.45 ± 17.56	185.69 ± 16.18	179.09 ± 29.60
Kidney/body weight (mg/g)	5.37 ± 0.66	8.80 ± 0.39	8.36 ± 1.39
Blood glucose (mg/dL)	181.20 ± 61.21	489.57 ± 144.00	437.17 ± 124.69
HbA1c (%)	5.92 ± 0.96	10.83 ± 1.43	9.60 ± 2.52
Serum bilirubin (mg/dL)	0.03 ± 0.02	0.07 ± 0.03*	0.04 ± 0.02^#^
Serum Creatinine (mg/dL)	0.18 ± 0.05	0.18 ± 0.05	0.13 ± 0.04
Serum BUN (mg/dL)	25.60 ± 5.37	36.43 ± 9.22	42.83 ± 17.02
Urine Creatinine (mg/dL)	50.42 ± 12.64	6.37 ± 0.47	10.46 ± 11.95
24-hr Urine Albumin (μg)	6.60 ± 2.73	49.99 ± 23.74	39.47 ± 26.10
UACR (μg/mg)	16.05 ± 8.47	37.06 ± 10.79*	31.60 ± 18.27^#^

STZ, streptozotocin; mPTD-BMP7, protein transduction domain-fused BMP7 in micelle; BUN, blood urea nitrogen; UACR, urine albumin-creatinine ratio. *P < 0.05; versus control group. ^#^P < 0.05; versus STZ + Vehicle group.

### 3.3 mPTD-BMP7 ameliorates ferroptotic damage to the kidney in diabetic conditions

**Figure 3:**
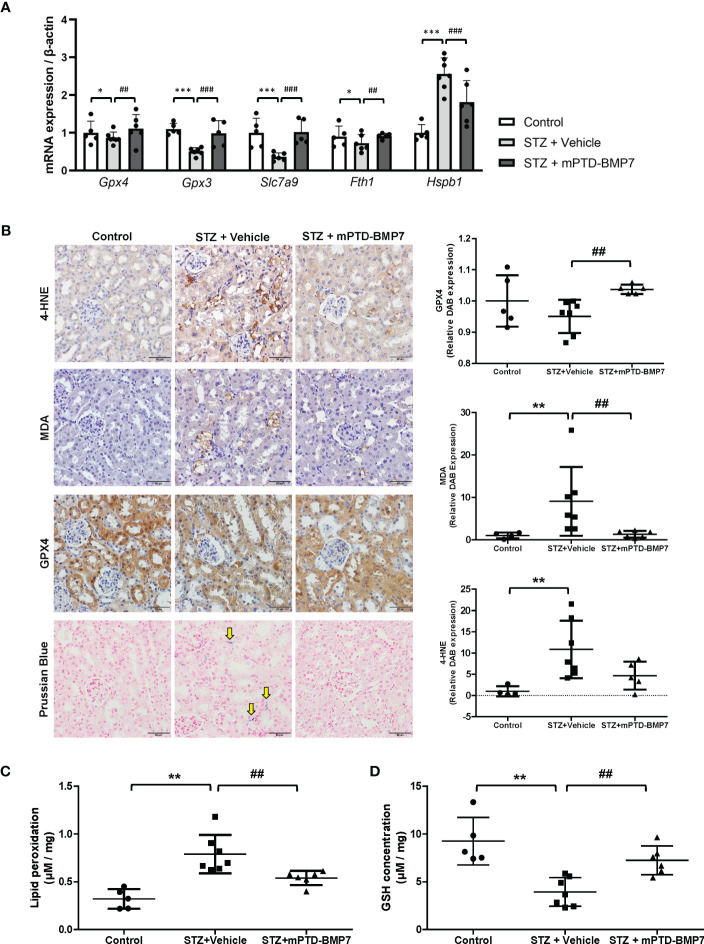
Effects of mPTD-BMP7 on ferroptosis in the kidneys of diabetic mice. **(A)** mRNA expression of ferroptosis-related genes significantly changed in the diabetic kidney, and the changes were abrogated by mPTD-BMP7. **(B)** Immunohistochemistry showed that damaged tubular cells exhibited increased level of 4-hydroxynonenal (4-HNE) and malondialdehyde (MDA) in the vehicle group, and it was abrogated by mPTD-BMP7 administration. Prussian blue staining revealed iron deposition in the interstitium of diabetic kidney (yellow arrow), and it was attenuated by mPTD-BMP7. Original magnification: x400 (scale bar: 50μm) **(C)** Lipid oxidation, assessed *via* MDA levels, was increased in the kidneys of STZ mice, and reduced by treatment with mPTD-BMP7. **(D)** GSH concentration in the kidneys of STZ mice was decreased, and it was attenuated by treatment with mPTD-BMP7. (*P < 0.05; **P < 0.01; ***P < 0.001 versus control group. ##P < 0.01; ###P < 0.001; versus mPTD-BMP7 group).

In the STZ-induced diabetic condition, renal tubular cells showed signs of cellular damage, but treatment with mPTD-BMP7 reduced damage. To confirm if ferroptotic damage had occurred in the kidneys of diabetic animals and was reduced by mPTD-BMP7 treatment, we evaluated key players in ferroptosis. mRNA levels of Gpx3, Gpx4, and Slc7a9 in diabetic kidney were significantly downregulated, but recovered after mPTD-BMP7 treatment (**Figure 3A**). Iron accumulation was confirmed in the diabetic kidney by Prussian blue staining and was prevented by mPTD-BMP7 (**Figure 3B**), consistent with significant change of mRNA expression levels of Fth1 and Hspb1 mRNA (**Figure 3A**). MDA and 4-HNE, the end products of lipid peroxidation, were significantly increased in the damaged tubular cells in diabetic kidney and markedly reduced by mPTD-BMP7 administration (**Figures 3B, C**). Additionally, reduced GSH concentration decreased in the diabetic kidney, but recovered after treatment with mPTD-BMP7 (**Figure 3D**). Based on these results, we confirmed that ferroptosis is a form of renal tubular damage induced by STZ and that mPTD-BMP7 can reduce this type of cellular damage.

### 3.4 Effect of mPTD-BMP7 on the progression of diabetic renal fibrosis

**Figure 4:**
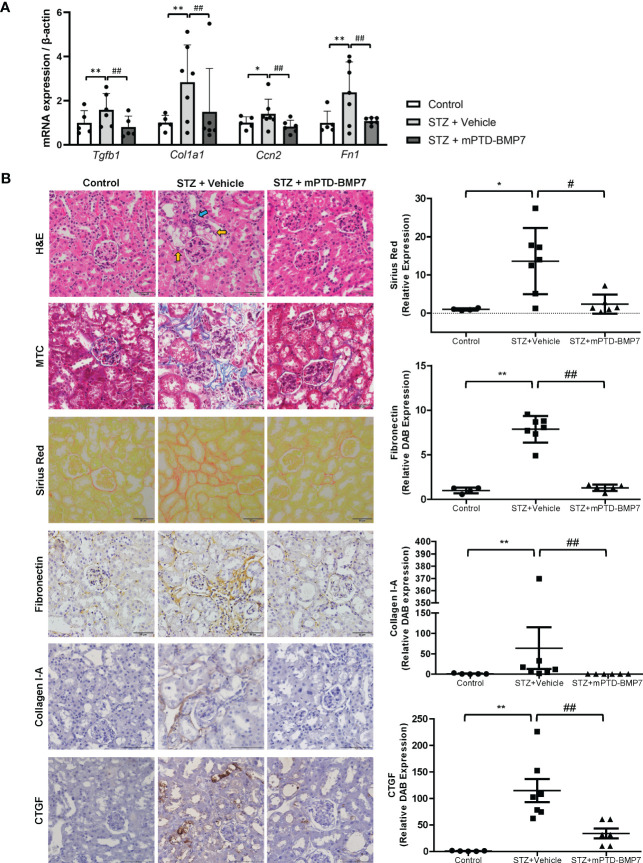
Effects of mPTD-BMP7 on fibrosis in the kidneys of diabetic mice. **(A)** Increased mRNA levels of fibrosis-related genes were attenuated by mPTD-BMP7 in the diabetic kidney. **(B)** Pathologic change in the diabetic kidney was observed in STZ-treated group, such as degeneration of tubular cells (yellow arrow) and tubulointerstitial inflammation (blue arrow) and fibrosis. It was abrogated by mPTD-BMP7 administration. Accumulation of collagen and fibronectin in the STZ-treated renal interstitium was detected in Masson’s trichrome (MTC), Sirius red staining and immunohistochemical staining of fibronectin and collagen type I-A, and it was reduced by mPTD-BMP2 administration. Original magnification: x400 (scale bar: 50μm). (*P < 0.05; **P < 0.01 versus control group. #P < 0.05; ##P < 0.01; versus mPTD-BMP7 group).

**Figure 5:**
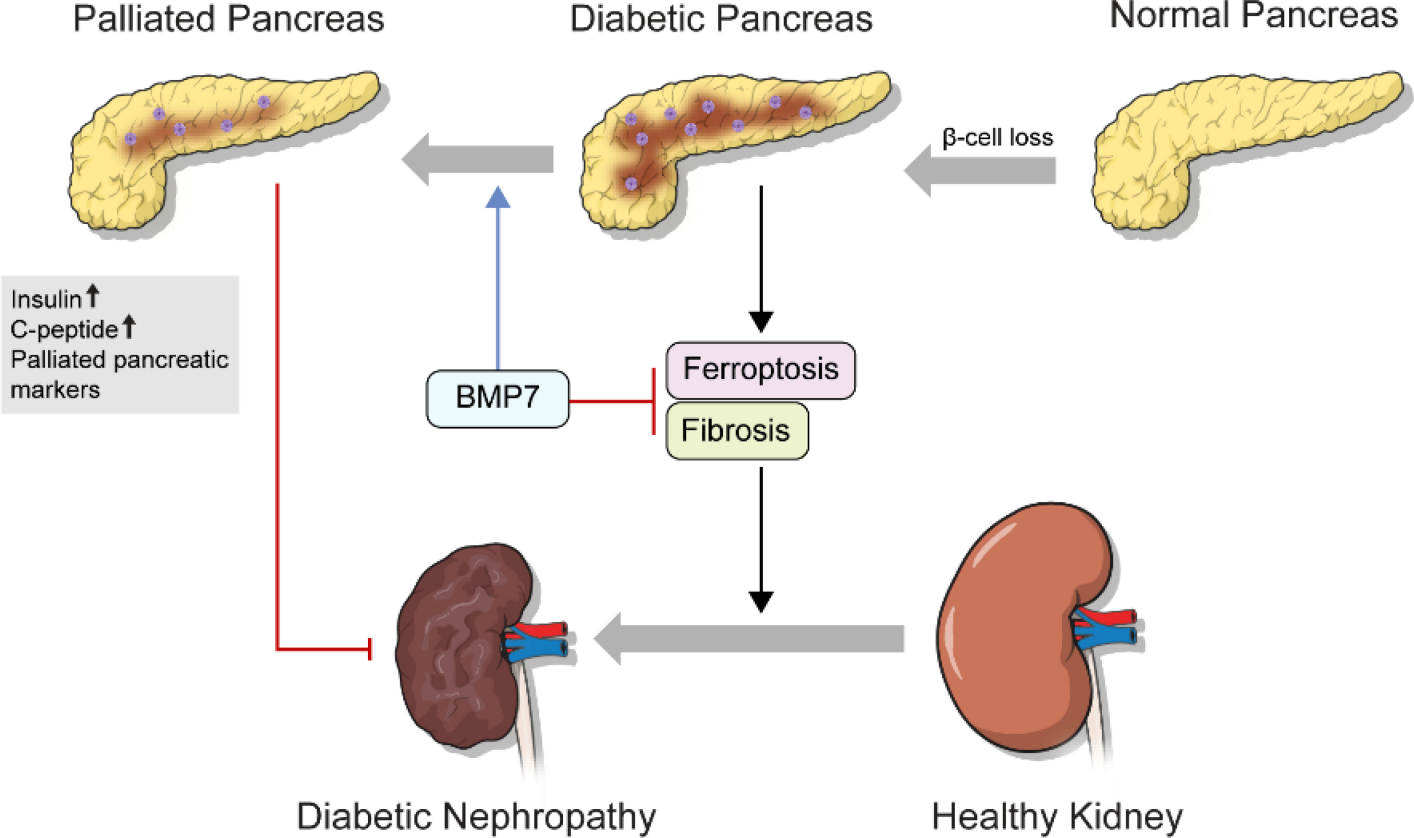
Schematic diagrams of the role of mPTD-BMP7 in the diabetic condition.

The STZ-induced diabetic kidney exhibited marked damage on renal cortical tubules, including tubular cell vacuolization, loss of nucleus, and structural destruction of tubule (**Figure 4B**). On the other hand, diabetic changes in the glomerulus, such as mesangial expansion and deposition of excessive extracellular matrix, were not marked. Most notably, tubulointersititial inflammation and fibrosis were observed in the STZ-treated group. However, administration of mPTD-BMP7 attenuated the cellular damage, tubulointerstitial inflammation and fibrosis (**Figure 4B**). The STZ-induced diabetic group exhibited a marked increase in Sirius red and Masson’s trichrome staining, as well as increased fibronectin, CTGF (connective tissue growth factor) and collagen type I-A expression on immunohistochemical staining, which are indications of fibrosis (**Figure 4B**). Also, the mRNA level of TGF-β1 was significantly increased in the diabetic kidney, and fibrosis-related downstream genes were upregulated. These upregulated transcriptions of TGF-β1 and fibrosis-related genes were hindered by mPTD-BMP7 (**Figure 4A**). Taken together, we confirmed that mPTD-BMP7 antagonized TGF-β1-induced fibrosis and ferroptotic damage in the STZ-induced diabetic nephropathy model (**Figure 5**).

## 4 Discussion

The loss or dysfunction of β cells is involved in the pathogenesis of most types of diabetes. Many factors induce β cell loss or dysfunction, such as genetic mutation, extrinsic metabolic stress, autoimmune antibodies, inflammation, pancreatitis, pancreatic cancer, and Alzheimer’s disease (26–28). Insulin-producing β cells can be generated from non-pancreatic somatic cells, pancreatic exocrine cells, or pancreatic islet cells by transdifferentiation (29). Many studies have reported obtaining β cells from murine hepatocytes by the overexpression of pancreatic transcript factors that are important for β cells such as PDX1, NEUROD1, and neurogenin-3 (Ngn3) *via* adenoviral delivery in diabetic animal models (30–33). Efficient protocols of gene therapy have been proposed to convert α-cells into β cells, where adenoviral delivery of transgenic constructs such as PDX1 and MAFA genes *via* pancreatic duct has proven to expand β-cells in STZ-treated and nonobese diabetic mice (34). Further, acinar cells, as a prospective source of β cell generation, are abundant, in close proximity to, and share developmental origin with endocrine cells (29). Overexpression of pancreatic transcription factors such as PDX1, Ngn-3, and MafA or exposure to cytokines and growth factors enables efficient transdifferentiation into β cells in rodent models and rodent cell experiments (35–37). While the genetic manipulations are interesting, druggable therapeutics for pancreatic regeneration are limited currently. Recently, the β cell-like phenotype was induced by treatment with BMP7 in human nonendocrine pancreatic tissue (38). Further, BMP7 stimulated pancreatic progenitor cell proliferation by binding to ALK3, which is co-expressed with PDX1 in multipotent cells in exocrine pancreas (39). In our diabetic pancreas model, we found that reduced expression of many adult pancreatic markers (INS, SYP, Slc2a1, and KRT19) and pancreatic developmental markers (Pdx1, Prox1, Hnf1b, and Neurod1) in STZ-induced diabetic model was attenuated by the intraperitoneal administration of mPTD-BMP7, assuming pancreatic exocrine-endocrine conversion has partially occurred. Considering the irreversible damage to the insulin-producing beta cells of the pancreas by STZ, our study suggests the regenerative potential of mPTD-BMP7 in treating pancreatic beta cells.

Chronic kidney disease (CKD) caused by DM is diagnosed as DN, with gradual kidney dysfunction *via* nephron loss and characteristic pathological findings, such as increased mesangial substrate, tubular cell death, nodular lesions, and tubulointerstitial fibrosis (40, 41). Not only microvascular and glomerular injuries but also tubular atrophy and tubular epithelial cell death play a role in this condition. Hyperglycemia triggers the generation of free radicals and oxidative stress in the tubular cells, mediating proliferation, extracellular matrix deposition, and cell death (42). Since the proximal tubule requires a supply of high-energy phosphate compounds and relies on aerobic metabolism, reduced oxygen supply to the metabolically active tubular epithelial cells makes them vulnerable to diabetic ischemic injury (42, 43). Tubular cell injury and death are key findings of kidney damage in diseases such as unilateral ureteral obstruction, AKI, renal ischemia followed by reperfusion, and CKD of diabetic conditions (44–56). In our STZ model, diabetic kidney exhibited damaged renal tubules and tubulointerstitial fibrosis, but less pathologic change in the glomerulus. In addition, damage to the kidneys showed regional and early-to-mid stage destruction rather than diffuse and end-stage destruction. Our 16-week animal experiment may have been insufficient to induce chronic severe renal destruction; this was not considered to cause serious deterioration in serum and urinary functional parameters.

Many studies have focused on the modalities of apoptosis and necrosis in kidney disease; recent advances have enhanced our understanding of novel concepts of regulated necrosis, such as necroptosis, ferroptosis, pyroptosis, and mitochondrial permeability transition-dependent regulated necrosis (42). In an STZ-induced DN model, damaged tubular cells undergo various types of programmed cell death; apoptosis, autophagy, necroptosis and ferroptosis (8, 14, 57–59). Several recent studies have tried to identify the role of ferroptosis in this DN model (14, 60–62). Unlike autophagy and apoptosis, ferroptosis is a distinct type of regulated cell death characterized by iron and O_2_-dependent accumulation of lipid hydroperoxides to lethal levels. It was first proposed by Dixon et al. as a type of cell death triggered by the depletion of cellular amino acid cysteine or by inhibition of GPX4, causing iron and O_2_-dependent accumulation of lipid hydroperoxides to lethal levels (10). Disruption of the intracellular thiol antioxidant network progresses to non-apoptotic, iron-dependent, oxidative cell death, termed ferroptosis (9). In many cells, GSH synthesis is dependent on the continuous import of cysteine by the cell surface Cys_2_/glutamate antiporter system x_c_^-^ (63, 64), the heterodimer of a membrane protein b^0,+^AT/SLC7A9, and its auxiliary subunit rBAT/SLC3A1, which is responsible for cysteine reabsorption, particularly in renal proximal tubules (65–68). Although Slc7a9 mutations have been reported to cause cystinuria, their functions in protecting proximal tubular cells against iron-induced oxidative damage (65, 66, 68) and ferroptosis are unclear. Slc7a9 showed a decreased expression in erastin- or TGF-β1-stimulated proximal tubular cells and in the kidneys of diabetic STZ mice. mPTD-BMP7 reversed this reduction in Slc7a9 expression. According to our results, Slc7a9 is assumed to be involved in ferroptosis as a novel cysteine importer in renal proximal tubular cells.

Among the factors that lead to the progression of DN, TGF-β1 is considered a master regulator of DN development in both type 1 and type 2 DM (4, 6, 7, 69). It is a pleiotropic cytokine involved in angiogenesis, immunomodulation, and ECM formation, stimulated by various factors including hyperglycemia and ROS in kidney cells (7). There is abundant evidence that TGF‐β plays a predominant role in kidney fibrosis, confirmed by the fact that suppression of the TGF‐β/Smad signaling pathway attenuates kidney fibrosis and injury (5). Recently, TGF‐β has been reported to induce ferroptosis by inhibiting GPX4 and cysteine transporters in hepatocellular carcinoma cells and renal tubular cells, enhancing lipid-oxidation (14, 70). Moreover, TGF‐β and ROS interact not only in their self-synthesis, but also in the downregulation of antioxidant enzymes such as glutaredoxin, catalase, superoxide dismutase, and GPX (71), the latter which has been proposed to alleviate the activation of the TGF‐β/SMAD signaling pathway, which is involved in the progression of myelofibrosis (72). In accordance with these findings, we demonstrated that Gpx4 was involved both in ferroptosis and fibrosis, and that its action was hindered by mPTD-BMP7.

Despite our results showing improvement of renal fibrosis and ferroptosis markers in tissue level and kidney functional parameters such as UACR by mPTD-BMP7, other parameters did not match the tissue status. Although a persistent decrease in eGFR (estimated glomerular filtration rate) or increase in UACR are the commonly accepted parameters in diagnosing diabetic nephropathy (73), GFR is not usually appropriate in laboratory animal model (74). However, other parameters such as BUN, serum creatinine, and urinary albumin secretion were not significantly improved. As seen in **Figure 2A**, the delivery of the mPTD-BMP7 was primarily concentrated in the pancreas and much less in kidney, suggesting that our form of BMP7 may not have been sufficient in kidney to significantly improve all renal functional parameters. Recently, we reported that interventional administration *via* renal artery in a large animal model revealed efficient delivery of mPTD-BMP7 into the kidney, resulting in inhibition of fibrotic progression of kidney (23). Therefore, peritoneal administration of mPTD-BMP7 in this study may have less therapeutic potential to the kidney.

Similarly, mPTD-BMP7 improves C-peptide and insulin in the STZ-induced mouse model, leaving blood glucose and HbA1c level. Considering the irreversible toxicity of STZ to beta cells and dietary control in mice, we believe that mPTD-BMP7 restores pancreatic damage from the STZ. Considering these together, BMP7 can be considered an alternative therapy against diabetes-induced damage to the pancreas and kidney. BMP7 helped prevent progression to DN *via* ferroptosis as well as *via* inhibiting the canonical TGF‐β signaling. Further pharmacologic and preclinical studies are required to identify clinical therapeutics for DM.

## Data availability statement

The original contributions presented in the study are included in the article/supplementary material. Further inquiries can be directed to the corresponding authors.

## Ethics statement

The animal study was reviewed and approved by Institutional Animal Care and Use Committee of the Yonsei University.

## Author contributions

SHS, DH performed experiments; KP, JU, and SK supported *in vitro* and *in vivo* experiments. MK and JY performed *in vivo* image analysis. T-HY, NK, JIY, and HK planned all experiments, analyzed the data, and wrote the manuscript. All authors contributed to the article and approved the submitted version.
